# Early goal-directed therapy after major surgery reduces complications and duration of hospital stay. A randomised, controlled trial [ISRCTN38797445]

**DOI:** 10.1186/cc3887

**Published:** 2005-11-08

**Authors:** Rupert Pearse, Deborah Dawson, Jayne Fawcett, Andrew Rhodes, R Michael Grounds, E David Bennett

**Affiliations:** 1 Adult Intensive Care Unit, 1st floor St James' Wing, St George's Hospital, Blackshaw Road, London SW17 0QT, UK

## Abstract

**Introduction:**

Goal-directed therapy (GDT) has been shown to improve outcome when commenced before surgery. This requires pre-operative admission to the intensive care unit (ICU). In cardiac surgery, GDT has proved effective when commenced after surgery. The aim of this study was to evaluate the effect of post-operative GDT on the incidence of complications and duration of hospital stay in patients undergoing general surgery.

**Methods:**

This was a randomised controlled trial with concealed allocation. High-risk general surgical patients were allocated to post-operative GDT to attain an oxygen delivery index of 600 ml min^-1 ^m^-2 ^or to conventional management. Cardiac output was measured by lithium indicator dilution and pulse power analysis. Patients were followed up for 60 days.

**Results:**

Sixty-two patients were randomised to GDT and 60 patients to control treatment. The GDT group received more intravenous colloid (1,907 SD ± 878 ml versus 1,204 SD ± 898 ml; *p *< 0.0001) and dopexamine (55 patients (89%) versus 1 patient (2%); *p *< 0.0001). Fewer GDT patients developed complications (27 patients (44%) versus 41 patients (68%); *p *= 0.003, relative risk 0.63; 95% confidence intervals 0.46 to 0.87). The number of complications per patient was also reduced (0.7 SD ± 0.9 per patient versus 1.5 SD ± 1.5 per patient; *p *= 0.002). The median duration of hospital stay in the GDT group was significantly reduced (11 days (IQR 7 to 15) versus 14 days (IQR 11 to 27); *p *= 0.001). There was no significant difference in mortality (seven patients (11.3%) versus nine patients (15%); *p *= 0.59).

**Conclusion:**

Post-operative GDT is associated with reductions in post-operative complications and duration of hospital stay. The beneficial effects of GDT may be achieved while avoiding the difficulties of pre-operative ICU admission.

## Introduction

Goal-directed therapy (GDT) is a term used to describe the use of cardiac output or similar parameters to guide intravenous fluid and inotropic therapy. When commenced in the pre-operative period, this technique has been shown to improve outcome after major general surgery [1-3]. Although the number of post-operative deaths has changed little in recent years [4,5], pre-operative GDT has not been introduced into routine practice. The principal reason for this is likely to be the limited availability of intensive care unit (ICU) facilities, but there are also safety concerns regarding the use of the pulmonary artery catheter to measure cardiac output [6].

In cardiac surgery, these problems have been addressed successfully by commencing GDT in the immediate post-operative period [7,8] and by using the oesophageal Doppler probe in place of the pulmonary artery catheter to measure cardiac output [8]. Use of the oesophageal Doppler probe to guide fluid administration during surgery is also associated with improved outcome [9-13] Unfortunately the Doppler probe is not readily tolerated by conscious patients, restricting use to patients who are ventilated following surgery.

There is a need to establish whether GDT is effective when commenced after major general surgery. This study was undertaken to assess the effect of post-operative GDT on complication rates and duration of hospital stay in high-risk general surgical patients.

## Materials and methods

### Participants

Adult patients scheduled for major general surgery and deemed to be at a high risk of post-operative complications were enrolled in accordance with criteria similar to those used in previous trials (see Additional file 1) [1,2]. Patients were screened for eligibility by a member of the research team, who obtained written informed consent before surgery. This study was approved by the Local Research Ethics Committee of St George's Healthcare National Health Service Trust.

### Protocol

**Figure 1:**
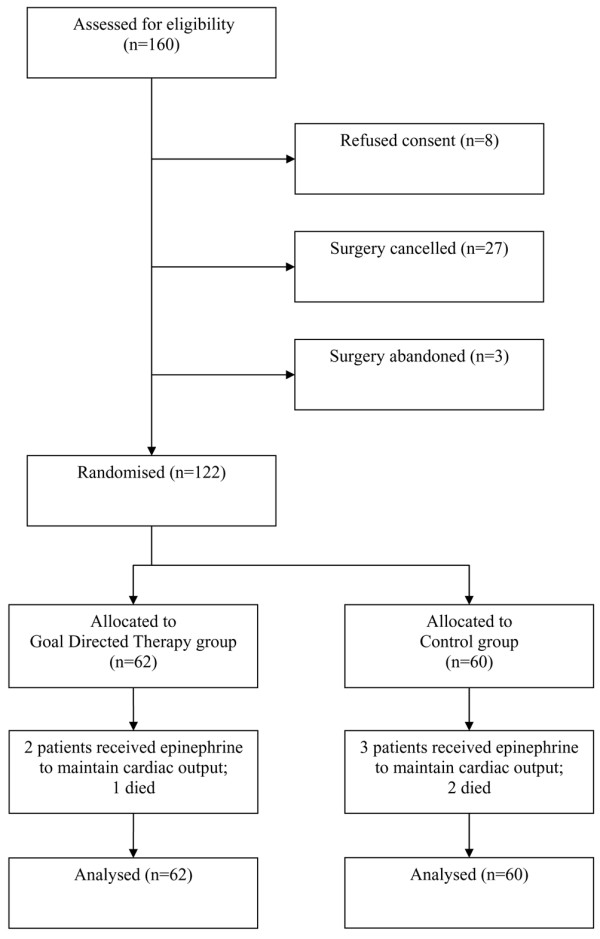
Flow of participants through the trial.

This was a randomised controlled, partly blind, single-centre study conducted in the adult ICU at St George's Hospital, London. The primary outcome measure was the incidence of post-operative complications. Secondary outcome measures were the duration of hospital stay and mortality. Patients were assigned to GDT or control groups by computer-generated random sequence. Study group assignments were placed in serially numbered opaque envelopes. Randomisation was performed by a member of the research team when surgery was complete. Data were analysed on an intention-to-treat basis, including all patients who were randomised (Figure 1).

**Figure 2:**
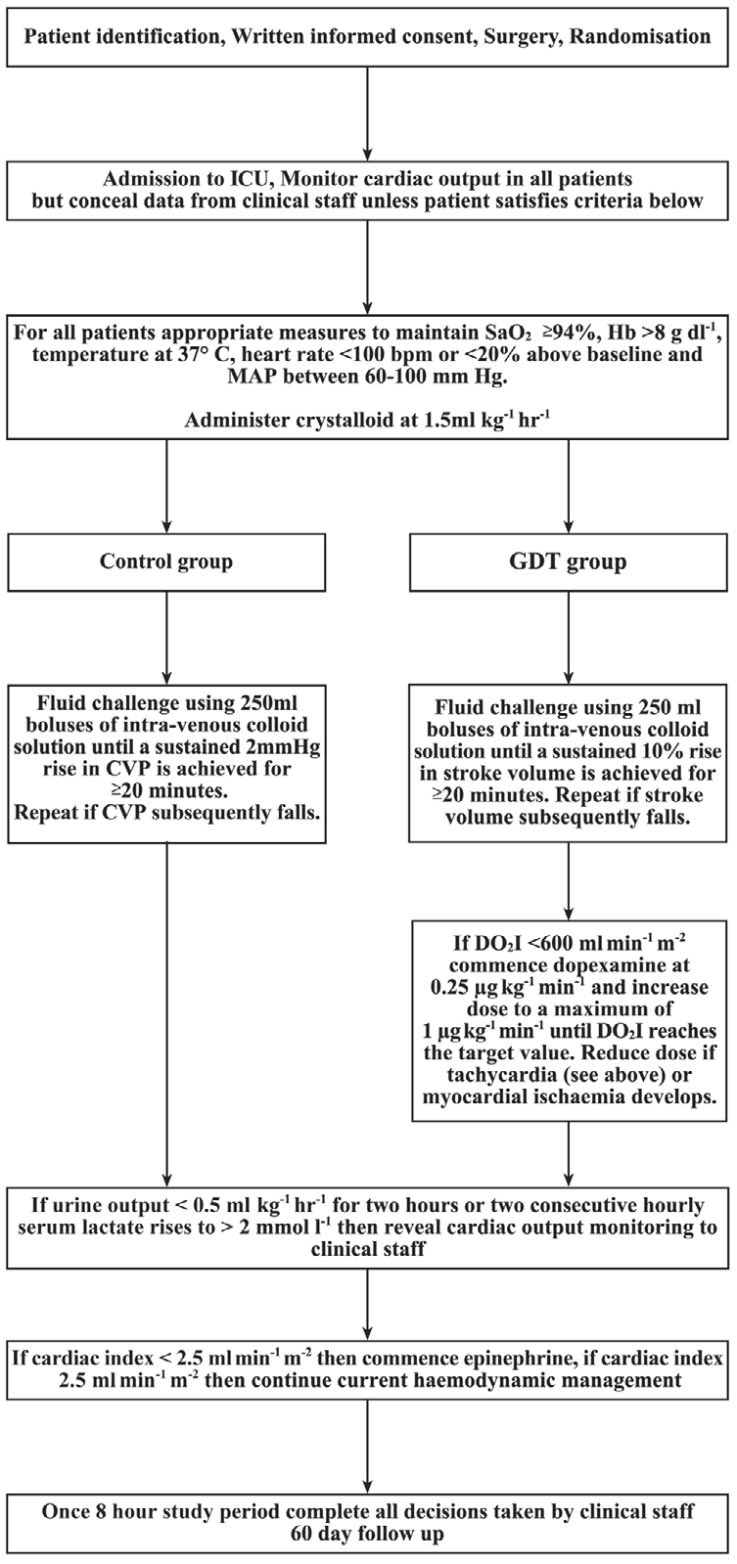
Cardiovascular treatment protocols for goal-directed therapy (GDT) and control groups. DO_2_I, oxygen delivery index; Hb, haemoglobin; SaO_2_, arterial oxygen saturation.

Protocols for haemodynamic management during the first 8 hours after surgery are summarised in Figure 2. Patients in the control group were administered 250 ml boluses of intravenous colloid solution (Gelofusine; B Braun Medical Ltd., Sheffield, UK) to achieve a sustained increase in central venous pressure (CVP) of at least 2 mmHg for 20 minutes. GDT patients received 250 ml boluses of intravenous colloid solution to achieve a sustained rise in stroke volume of at least 10% for 20 minutes. Fluid challenges were repeated if the target parameter subsequently decreased or if there was strong clinical suspicion of persistent hypovolaemia. The GDT group also received dopexamine up to a maximum of 1 μg kg^-1 ^min^-1 ^if oxygen delivery index (DO_2_I) did not reach 600 ml min^-1 ^m^-2 ^with intravenous fluid alone. The dose of dopexamine was reduced or discontinued in patients who became tachycardic (heart rate above 100 beats min^-1 ^or an increase greater than 20% above baseline) or developed myocardial ischaemia (clinical symptoms or electrocardiograph criteria). These treatments were administered by a member of the research team who was the only individual aware of study group allocation. All other aspects of patient care were handled by clinical staff. Dopexamine was prepared in a masked syringe for each patient in the GDT group, while a dummy infusion (normal saline) was prepared in a similarly masked syringe for all patients in the control group. Cardiac output data were concealed from non-research staff unless predefined criteria were satisfied allowing the use of cardiac output data in deteriorating patients (Figure 2).

### Assessments

The following parameters were monitored continuously during the study period: electrocardiograph, pulse oximetry, invasive arterial pressure, CVP and cardiac output. Lithium indicator dilution and pulse power analysis was used to measure cardiac output and to calculate DO_2_I (LiDCO plus system; LiDCO Ltd., Cambridge, UK). This technique is minimally invasive and well validated [14]. Arterial blood gas measurements were performed hourly during the study period. P-POSSUM (Portsmouth Physiologic and Operative Severity Score for the enUmeration of Mortality and morbidity) and APACHE II (Acute Physiology and Chronic Health Evaluation II) scores were calculated after admission to the ICU [15,16]. Patients were followed up for 60 days. Diagnosis and management of complications were undertaken by non-research staff. These were verified, in accordance with predefined criteria, by a member of the research team unaware of study group allocation. This process involved inspection of notes, radiological investigations, laboratory data and clinical assessment.

### Statistical analysis

Assuming a two-sided type I error rate of 5% and a power of 80%, we calculated that a sample size of 300 patients would be required to detect a reduction in the proportion of patients developing complications from 50% in the control group to 34% in the GDT group. These values were based on the observed incidence of complications in the control groups of previous similar trials [1,3]. Arrangements were made prospectively for interim analyses after the recruitment of 100 and 200 patients. To minimise the possibility of type I error at interim analysis, a more stringent level of significance was required (*p *< 0.01) for the trial to be stopped after interim analysis than that used for the initial power analysis (*p *< 0.05).

Data are presented as means (standard deviation) where normally distributed, and as median (interquartile range) where not normally distributed. Relative risk is presented with 95% confidence intervals. Categorical data were tested with Fisher's exact test. Continuous data were tested with the *t *test where normally distributed, and with the Mann–Whitney *U *test where not normally distributed. Confidence intervals were constructed for the difference in mean duration of stay between the two groups by bootstrapping within treatment groups [17]. Analysis was performed with GraphPad Prism version 4.0. Significance was set at *p *< 0.05.

## Results

A total of 122 patients were recruited between November 2002 and August 2004 (Figure 1). The study was stopped early on the advice of the external safety assessor, after assessment of data from the first 100 patients, because the primary end-point had been achieved. By this time 62 patients had been randomised to the GDT group and 60 patients to the control group. The groups were well matched for age, sex, blood loss, type of surgery and anaesthetic technique (Table 1).

**Figure 3:**
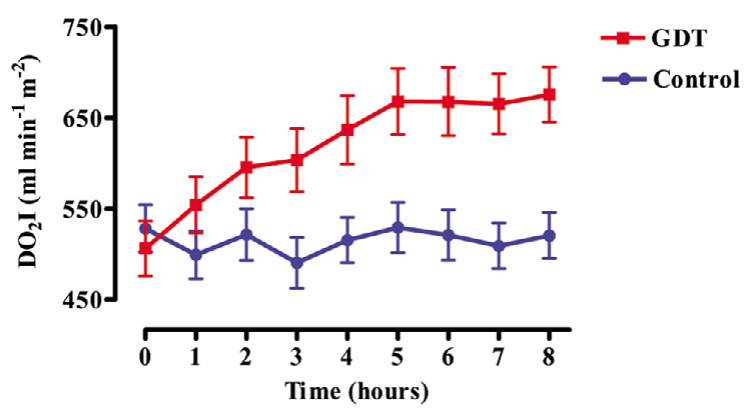
Oxygen delivery index for goal-directed therapy and control groups during the 8-hour study period. Results are means ± SEM. DO_2_I, oxygen delivery index; GDT, goal-directed therapy.

The goal for DO_2_I was achieved by most patients in the GDT group and spontaneously by a smaller proportion of the control group (49 patients (79%) versus 27 patients (45%); *p *= 0.0002; Figure 3). Patients in the GDT group received a greater volume of colloid solution but a similar volume of blood (Table 2). Dopexamine was administered to 55 patients in the GDT group and, on the instruction of clinical staff, to one patient in the control group. Despite receiving the maximum therapy allowed by the protocol, 13 patients in the GDT group did not achieve the goal for DO_2_I. In seven of these patients the dose of dopexamine was reduced either because of tachycardia (six patients) or myocardial ischaemia (one patient).

Fewer patients developed complications in the GDT group (27 patients (44%) versus 41 patients (68%); relative risk 0.63; 95% confidence interval 0.46 to 0.87; *p *= 0.003). The total number of complications per patient was also lower in the GDT group (0.7 per patient (SD 0.9) versus 1.5 per patient (SD 1.5); *p *= 0.002; Tables 3 and 4). The reduction in the number of post-operative complications in the GDT group was associated with a reduction in both mean duration of hospital stay (17.5 days versus 29.5 days, 41% reduction (95% confidence intervals 0 to 81); *p *= 0.001) and median duration of stay (11 days (7 to 15) versus 14 days (11 to 27); *p *= 0.001). There was no difference in duration of ICU stay (43 hours (24 to 102) versus 45 hours (25 to 99); *p *= 0.82). There were no delays in discharge as a result of problems in organising nursing home placement or other social care. There were no significant differences in 28-day or 60-day mortality (Table 3). The 28-day mortality predicted with the P-POSSUM score was higher for the GDT than for the control group (18.5% versus 13.7%; *p *= 0.09).

## Discussion

This is the first study to investigate the effects of post-operative GDT in high-risk patients undergoing major general surgery. The effect of the GDT protocol was to reduce the number of patients developing complications and shorten their hospital stay in comparison with a protocol designed to reflect standard care. Thus, some of the beneficial effects of GDT might still be achieved when pre-operative ICU admission is not possible. In addition, the use of lithium indicator dilution and pulse power analysis to measure cardiac output obviates the need for insertion of a pulmonary artery catheter. The GDT protocol used in this study is therefore a practical and effective intervention.

The mortality in both groups was lower than had been predicted by the P-POSSUM score, suggesting that all patients received a high standard of care. All patients in the control group were admitted to the ICU and received intravenous fluid resuscitation guided by CVP measurements. Rather than apply an absolute target for CVP, a dynamic fluid response target of at least 2 mmHg was used. This provides a more reliable guide to fluid requirements and avoids discrepancies arising from differences in intrathoracic pressures between patients who are ventilated and those who are not. In addition, cardiac output was measured in all patients and was revealed to clinical staff according to predefined criteria. It is difficult to design a simple protocol that will account for all eventualities. Allowance was therefore made for the administration of a fluid challenge where there was strong clinical suspicion of hypovolaemia, but a fluid challenge was not mandated by the protocol.

In several studies, GDT has been shown to improve outcome when commenced before surgery [1-3]. However, a recent multi-centre trial that randomised surgical patients to pulmonary artery catheterisation or conventional management failed to show a difference in outcome [18]. These findings might have occurred as a result of several important methodological flaws, which have been discussed elsewhere [19]. The important differences between the present study and previous work in general surgical patients are that the protocol was commenced after surgery, was only 8 hours in duration and did not require the use of a pulmonary artery catheter. This design is similar to two successful trials of post-operative GDT in cardiac surgical patients [7,8]. A recent retrospective study illustrates the continued interest in the use of peri-operative β-blockade in high-risk surgical patients [20], although the results of a multi-centre trial are still awaited [21]. The apparent efficacy of GDT and β-blockade relates to effects on different pathophysiological processes. Both treatments are likely to have a role in the management of the high-risk surgical patient.

The mechanism of the therapeutic effect of GDT remains unclear. It may be that increased global oxygen delivery results in increased tissue partial pressure of oxygen (PO_2_), with improved tissue healing and reduced infection rates. There is some evidence that additional intravenous fluid use improves tissue PO_2 _during surgery [22] and that decreases in global oxygen delivery and regional oxygen tension are associated with poor tissue healing and infection [23,24]. The use of GDT may also have financial implications. Previous studies have shown peri-operative GDT to be associated with an overall cost reduction [25,26]. In the present study, GDT was associated with a 41% reduction in mean duration of hospital stay. This suggests that the use of GDT might reduce the overall cost of surgical care.

There are some potential weaknesses in the design of this study. It was a small single-centre study that was stopped early after interim analysis of a composite end-point. These factors limit the applicability of the findings. Recruitment was possible only when a member of the research team was available to take informed consent before surgery and administer the 8-hour study protocol. During the trial period, 979 surgical patients were admitted to ICU, with a hospital mortality of 12.1%. Not all of these patients would have been eligible for recruitment. Studies of any interventional protocol will require at least one individual to be aware of study group allocation. Although this does increase the possibility of bias, we took several measures to conceal allocation from everyone except the member of the research team delivering the protocol. Masked infusions and identical monitoring equipment were used for all patients. It would not have been possible for non-research staff to identify study group allocation.

## Conclusion

The use of post-operative GDT is associated with reductions in complications and duration of hospital stay but avoids the problems associated with pre-operative ICU admission and pulmonary artery catheterisation. A large multi-centre trial should be performed to validate the applicability of these findings to a wider population.

## Key messages

• 	Goal Directed Therapy has been shown to improve outcome when commenced before surgery, but this approach has proved impractical.

• 	This study suggests that post-operative Goal Directed Therapy is also effective, but does not require pre-operative ICU admission.

## Abbreviations

CVP = central venous pressure; DO_2_I = oxygen delivery index; GDT = goal-directed therapy; ICU = intensive care unit; IQR = Interquartile range; PO_2 _= partial pressure of oxygen; P-POSSUM = Portsmouth Physiologic and Operative Severity Score for the enUmeration of Mortality and morbidity.

## Competing interests

RP received a travel grant from LiDCO Ltd. to present this data at an international meeting. JF has previously performed consultancy work for LiDCO Ltd. DB currently performs consultancy work for LiDCO Ltd. and has previously performed consultancy work for Deltex Ltd. No other competing interests are declared.

## Authors' contributions

RP, DD, AR, MG and DB were responsible for study design. RP, DD and JF were responsible for administering the protocol. All authors were involved in data analysis and drafting the manuscript and approved the final version. All authors had full access to data and take responsibility for the integrity of the data and the accuracy of the analysis.

## Supplementary Material

Additional File 1 A Word file containing the admission and exclusion criteria for this study. Click here for file

## Figures and Tables

**Table 1:** Baseline characteristics of patients in the goal-directed therapy (GDT) and control groups

Characteristics	Control group	GDT group
Number in group	60	62
Age (years)	68 ± 11.6	66 ± 11.4
Male	39 (65%)	42 (68%)
P-POSSUM operative score	17.1 ± 4.9	17.8 ± 4.9
P-POSSUM physiology score	19.8 ± 5.5	21.9 ± 6.7
APACHE II score	9.6 ± 4.3	9.4 ± 3.9
ASA status 3 and above	43 (72%)	50 (81%)
Aged over 70 years with limited physiological reserve	20 (33%)	13 (21%)
Known history of severe cardiac or respiratory illness	22 (37%)	19 (31%)
Extensive surgery planned for carcinoma involving bowel anastomosis	21 (35%)	27 (44%)
Late-stage vascular disease involving the aorta	15 (25%)	11 (18%)
Elective surgery	54 (90%)	55 (88%)
Type of surgery		
Vascular	16 (27%)	12 (19%)
Upper gastro-intestinal	8 (13%)	16 (26%)
Hepato-biliary	4 (7%)	8 (13%)
Lower gastro-intestinal	14 (23%)	10 (16%)
Urology	15 (25%)	11 (18%)
Other	3 (5%)	5 (8%)

Values are absolute (%) or mean ± SD. APACHE, Acute Physiology and Chronic Health Evaluation; ASA, American Society of Anesthesiologists; P-POSSUM, Portsmouth Physiologic and Operative Severity Score for the enUmeration of Mortality and morbidity.

**Table 2:** Therapeutic interventions and changes in physiological parameters during the 8-hour study period

Parameter	Control group	GDT group	*p*
Intervention			
Crystalloid (ml)	960 ± 335	930 ± 221	0.39
Colloid (ml)	1,204 ± 898	1,907 ± 878	<0.0001
Blood (ml)	0 (0–485)	125 (0–734)	0.10
Dopexamine (μg kg^-1 ^min^-1^)	0.0 (0.0–0.0)	0.75 (0.5–1.0)	<0.0001
Change in variable			
DO_2_I (ml min^-1 ^m^-2^)	-10 ± 190	+163 ± 249	0.0001
Stroke volume (ml)	-7.2 ± 28.7	+6.7 ± 26.1	0.01
CVP (mmHg)	-1.6 ± 6.8	-2.1 ± 5.4	0.69
Serum lactate (mmol l^-1^)	-0.6 ± 1.1	-0.3 ± 0.8	0.11

Data are presented as mean ± SD or median (interquartile range). CVP, central venous pressure; DO_2_I, oxygen delivery index. GDT, goal-directed therapy.

**Table 3:** Summary of outcomes after 8-hour intervention period

Outcome measure	Control group	GDT group	*p*
Number of patients with complications	41 (68%)	27 (44%)	0.007
Median duration of hospital stay (days)	14 (11–27)	11 (7–15)	0.001
Mean duration of hospital stay (days)	29.5 (34.8)	17.5 (20.8)	0.001
Duration of ICU stay (hours)	45 (25–99)	43 (24–102)	0.82
28-day mortality	7 (11.7%)	6 (9.7%)	0.78
60-day mortality	9 (15%)	7 (11.3%)	0.59

Data are presented as median (interquartile range) or absolute value (%). ICU, intensive care unit. GDT, goal-directed therapy.

**Table 4:** Post-operative complications in goal-directed therapy (GDT) and control groups

Complication	Control group	GDT group
Infection		
Pneumonia	20	11
Abdominal	5	4
Urinary tract	3	1
Central venous catheter	6	2
Wound	20	4
Respiratory		
Pleural effusion	4	1
Pneumothorax	1	0
Pulmonary embolism	1	0
Acute respiratory distress syndrome	2	2
Cardiovascular		
Arrhythmia	9	5
Acute pulmonary oedema	4	3
Acute myocardial infarction	3	0
Stroke	1	2
Abdominal		
*Clostridium difficile *diarrhoea	1	0
Acute bowel obstruction	1	1
Upper gastro-intestinal bleed	4	2
Prolonged paralytic ileus	3	2
Anastomotic leak	1	1
Intra-abdominal hypertension	1	0
Post-operative massive haemorrhage	1	1
Total	90	43

## References

[B1] Boyd O, Grounds RM, Bennett ED (1993). A randomized clinical trial of the effect of deliberate perioperative increase of oxygen delivery on mortality in high-risk surgical patients. JAMA.

[B2] Shoemaker WC, Appel PL, Kram HB, Waxman K, Lee TS (1988). Prospective trial of supranormal values of survivors as therapeutic goals in high-risk surgical patients. Chest.

[B3] Wilson J, Woods I, Fawcett J, Whall R, Dibb W, Morris C, McManus E (1999). Reducing the risk of major elective surgery: randomised controlled trial of preoperative optimisation of oxygen delivery. BMJ.

[B4] Cullinane M, Gray AJ, Hargraves CM, Lansdown M, Martin IC, Schubert M (2003). The 2003 Report of the National Confidential Enquiry into Peri-Operative Deaths.

[B5] Campling EA, Devlin HB, Lunn JN (1990). Report of the National Confidential Enquiry into Peri-Operative Deaths.

[B6] Soni N (1996). Swan song for the Swan-Ganz catheter?. BMJ.

[B7] Polonen P, Ruokonen E, Hippelainen M, Poyhonen M, Takala J (2000). A prospective, randomized study of goal-oriented hemodynamic therapy in cardiac surgical patients. Anesth Analg.

[B8] McKendry M, McGloin H, Saberi D, Caudwell L, Brady AR, Singer M (2004). Randomised controlled trial assessing the impact of a nurse delivered, flow monitored protocol for optimisation of circulatory status after cardiac surgery. BMJ.

[B9] Gan TJ, Soppitt A, Maroof M, el-Moalem H, Robertson KM, Moretti E, Dwane P, Glass PS (2002). Goal-directed intraoperative fluid administration reduces length of hospital stay after major surgery. Anesthesiology.

[B10] Mythen MG, Webb AR (1995). Perioperative plasma volume expansion reduces the incidence of gut mucosal hypoperfusion during cardiac surgery. Arch Surg.

[B11] Sinclair S, James S, Singer M (1997). Intraoperative intravascular volume optimisation and length of hospital stay after repair of proximal femoral fracture: randomised controlled trial. BMJ.

[B12] Venn R, Steele A, Richardson P, Poloniecki J, Grounds M, Newman P (2002). Randomized controlled trial to investigate influence of the fluid challenge on duration of hospital stay and perioperative morbidity in patients with hip fractures. Br J Anaesth.

[B13] Wakeling HG, McFall MR, Jenkins CS, Woods WG, Miles WF, Barclay GR, Fleming SC (2005). Intraoperative oesophageal Doppler guided fluid management shortens postoperative hospital stay after major bowel surgery. Br J Anaesth.

[B14] Pearse RM, Ikram K, Barry J (2004). Equipment review: an appraisal of the LiDCO plus method of measuring cardiac output. Crit Care.

[B15] Knaus WA, Draper EA, Wagner DP, Zimmerman JE (1985). APACHE II: a severity of disease classification system. Crit Care Med.

[B16] Prytherch DR, Whiteley MS, Higgins B, Weaver PC, Prout WG, Powell SJ (1998). POSSUM and Portsmouth POSSUM for predicting mortality. Physiological and Operative Severity Score for the enUmeration of Mortality and morbidity. Br J Surg.

[B17] Thompson SG, Barber JA (2000). How should cost data in pragmatic randomised trials be analysed?. BMJ.

[B18] Sandham JD, Hull RD, Brant RF, Knox L, Pineo GF, Doig CJ, Laporta DP, Viner S, Passerini L, Devitt H (2003). A randomized, controlled trial of the use of pulmonary-artery catheters in high-risk surgical patients. N Engl J Med.

[B19] De Backer D, Creteur J, Vincent JL (2003). Perioperative optimization and right heart catheterization: what technique in which patient?. Crit Care.

[B20] Lindenauer PK, Pekow P, Wang K, Mamidi DK, Gutierrez B, Benjamin EM (2005). Perioperative beta-blocker therapy and mortality after major noncardiac surgery. N Engl J Med.

[B21] Devereaux PJ, Yusuf S, Yang H, Choi PT, Guyatt GH (2004). Are the recommendations to use perioperative beta-blocker therapy in patients undergoing noncardiac surgery based on reliable evidence?. CMAJ.

[B22] Arkilic CF, Taguchi A, Sharma N, Ratnaraj J, Sessler DI, Read TE, Fleshman JW, Kurz A (2003). Supplemental perioperative fluid administration increases tissue oxygen pressure. Surgery.

[B23] Kusano C, Baba M, Takao S, Sane S, Shimada M, Shirao K, Natsugoe S, Fukumoto T, Aikou T (1997). Oxygen delivery as a factor in the development of fatal postoperative complications after oesophagectomy. Br J Surg.

[B24] Sheridan WG, Lowndes RH, Young HL (1987). Tissue oxygen tension as a predictor of colonic anastomotic healing. Dis Colon Rectum.

[B25] Guest JF, Boyd O, Hart WM, Grounds RM, Bennett ED (1997). A cost analysis of a treatment policy of a deliberate perioperative increase in oxygen delivery in high risk surgical patients. Intensive Care Med.

[B26] Fenwick E, Wilson J, Sculpher M, Claxton K (2002). Pre-operative optimisation employing dopexamine or adrenaline for patients undergoing major elective surgery: a cost-effectiveness analysis. Intensive Care Med.

