# Supplementary material for: Early goal-directed therapy after major surgery reduces complications and duration of hospital stay. A randomised, controlled trial [ISRCTN38797445]
Source: Crit Care. 2005 Nov 8;9(6):R687–93. doi: 10.1186/cc3887 (PMC1414018; doi:10.1186/cc3887)
Supplement: Additional File 1 — A Word file containing the admission and exclusion criteria for this study. [file cc3887-S1.doc]

# Additional file 1

##### Admission criteria

Patients aged 18 years or older presenting for major surgery expected to last more than one and a half hours were eligible for inclusion if one or more of the following criteria were satisfied before surgery:

1. Severe cardiac or respiratory illness resulting in severe functional limitation
2. Extensive surgery planned for carcinoma involving bowel anastamosis
3. Acute massive blood loss (>2.5 litres)
4. Aged over 70 years with moderate functional limitation of one or more organ systems
5. Septicaemia (positive blood cultures or septic focus)
6. Respiratory failure (PaO_2_ < 8 kPa on FiO_2_ >0.4 *i.e.* PaO_2_:FiO_2_ ratio < 20 kPa or ventilation >48hours)
7. Acute abdominal catastrophe (*e.g.* pancreatitis, perforated viscous, gastro-intestinal bleed)
8. Acute renal failure (urea >20 mmol l^-1^, creatinine >260 µ mol l^-1^)
9. Surgery for abdominal aortic aneurysm

#### Exclusion criteria

Refusal of consent, pregnancy, acute myocardial ischaemia prior to enrolment, patients receiving palliative treatment only, disseminated malignancy, patients unlikely to survive more than 6 hours, patients requiring intervention outside ICU within the first 6 hours following surgery, patients on lithium therapy, weight less than 40 kg.
